# The Significance of Emergency Surgical Operations for Severe Elderly Patients Considering Medical Costs and Activities of Daily Life

**DOI:** 10.7759/cureus.93963

**Published:** 2025-10-06

**Authors:** Kiyohiro Oshima, Yusuke Sawada, Yuta Isshiki, Yumi Ichikawa, Kazunori Fukushima, Yuto Aramaki, Kei Kawano, Mizuki Mori

**Affiliations:** 1 Department of Emergency Medicine, Gunma University Graduate School of Medicine, Maebashi, JPN

**Keywords:** activity of daily life, elderly people, emergency surgery, intensive care, medical cost

## Abstract

Purpose

The global population is aging rapidly, and physicians increasingly face challenges in determining the appropriateness of emergency surgery for elderly patients. This study aimed to evaluate the clinical significance of emergency operations in elderly patients.

Methods

This retrospective clinical study included patients transferred to the emergency department of Gunma University Hospital and admitted to the intensive care unit following emergency surgery between January 2013 and December 2019. Patients were categorized into three age groups: 18-64 years (Group Y), 65-84 years (Group M), and ≥85 years (Group E). Clinical courses, including activities of daily living (ADL), were compared across groups. The primary outcomes were hospital mortality and the proportion of patients discharged directly home; secondary outcomes included additional clinical characteristics.

Results

A total of 84 patients were included: 30 in Group Y, 42 in Group M, and 12 in Group E. Both preoperative and postoperative ADL scores were significantly lower in Group E. Medical costs did not differ significantly among the groups. Although hospital mortality was highest in Group E, the difference was not statistically significant. However, the proportion of patients discharged directly home was significantly lower in Group E.

Conclusions

Emergency operations in patients aged ≥85 years may achieve outcomes comparable to those in younger patients; however, the total treatment period is likely to be prolonged due to significantly reduced postoperative ADL.

## Introduction

The global population is aging rapidly across both developed and developing countries [1]. In the United States, individuals aged 85 and older represent the fastest-growing demographic, with their numbers projected to double by 2036 and triple by 2049. By 2050, 4.5% of the United States population will be aged 85 and above, up from 2.5% in 2030 [2]. Similarly, in Japan, the number of people aged 85 and over, approximately 6.1 million in 2020, is expected to nearly double to about 11.7 million by 2060 [3]. This indicates that Japan shares a similar aging trend with the United States.

Against this backdrop, a growing number of older adults are being admitted to EDs requiring urgent or emergent surgical care [4, 5]. Consequently, physicians are increasingly faced with difficult decisions regarding the appropriateness of emergency surgery for super-elderly patients, a significant and pressing issue in current clinical practice. Moreover, physicians must evaluate the benefits and risks of emergency surgical procedures for these patients from multiple perspectives, including success rates, prognosis, and healthcare costs. This is essential, as elderly patients undergoing surgery often experience poorer outcomes, with higher risks of perioperative or postoperative mortality, postoperative complications, extended hospital stays, or discharge to settings other than their own home (i.e., the need for rehabilitation, care, or nursing home placement) [6].

This study evaluated the clinical courses and medical costs of super-elderly patients who underwent emergency surgery and required postoperative intensive care, compared with those of younger patients. The aim was to assess the significance of emergency surgical intervention in super-elderly individuals.

## Materials and methods

This retrospective clinical study was approved by the Research Ethics Board of Gunma University Hospital (HS2020-005), with a waiver of informed consent. The study was publicly disclosed on the Gunma University website, and all medical records were completely de-identified.

Patients transferred to the ED of Gunma University Hospital and admitted to the ICU following emergency abdominal, pulmonary, or vascular surgery under general anesthesia between January 2013 and December 2019 were included. Eligible patients were aged 18 years or older, and both endogenous and exogenous causes were considered. Exclusion criteria included out-of-hospital cardiac arrest, absence of active treatment requests from family, terminal-stage malignant disease or liver cirrhosis, and cases initially admitted to general wards and later transferred to the ICU due to clinical deterioration. Emergency procedures such as transarterial embolization, esophagogastroduodenoscopy for hemostasis, endoscopic retrograde cholangiopancreatography, percutaneous catheter interventions for acute coronary syndrome or cerebral infarction, and cases involving brain injury were also excluded.

Patients were categorized into three age groups: 18-64 years (Group Y), 65-84 years (Group M), and ≥85 years (Group E). This classification reflects standard definitions, with individuals aged ≥65 generally considered elderly [7, 8]. Additionally, the average life expectancy in developed countries exceeds 80 years, 84.1 in Japan, 83.7 in Switzerland, and 83.2 in Spain as of 2020 [9]. The proportion of individuals aged 85 and older is projected to rise in the coming years.

The primary outcomes were hospital mortality and the proportion of patients discharged directly to their homes. Secondary outcomes included clinical course variables such as ICU and hospital lengths of stay, pre- and postoperative ADL assessed using the Barthel Index (BI) [10] (BI can be calculated using the website: https://www.mdcalc.com/calc/3912/barthel-index-activities-daily-living-adl), sequential organ failure assessment (SOFA) scores [11] (permission for use of the SOFA score was obtained from Springer Nature, License Number: 6095630320136) upon hospital arrival and prior to ICU admission, and medical costs incurred during hospitalization. These outcomes were compared across the three age groups.

Statistical analysis

Normality was assessed using the Shapiro-Wilk test. Variables were expressed as medians and interquartile ranges. Categorical variables were reported as counts and/or percentages. The Kruskal-Wallis test was used to compare continuous variables among the three groups, followed by post hoc analyses for group-wise differences. Categorical variables were compared using the chi-squared test. Paired t-tests were applied for comparisons between two corresponding groups. A p-value < 0.05 was considered statistically significant. Statistical analyses were performed using the SPSS (version 28.0; IBM Corp., Armonk, NY, USA).

## Results

Eighty-four patients were transferred to the ED of our hospital and admitted to the ICU following emergency surgery between January 2013 and December 2019. Of these, 30 were in Group Y, 42 in Group M, and 12 in Group E.

Table 1 presents the patient characteristics across the three groups. Significant differences were observed in SOFA scores upon ED arrival and intraoperative blood loss. However, no significant differences were found in anesthesia time, operation time, or intraoperative blood transfusion volume. SOFA scores at ICU admission were ultimately similar among the groups, with no significant differences.

**Table 1:** Characteristics of patients in the three groups. ※1: p=0.0000000000000012212, ※2: p=0.00004424. SOFA: Sequential Organ Failure Assessment.

Variable	Group Y (n = 30)	Group M (n = 42)	Group E (n = 12)	Test statistic	p-value
Age (years) (median (Q1, Q3))	54 (42, 60)	67 (54, 74)	88 (86, 89)	68.676 (Kruskal-Wallis test)	※1
Male/female ratio	17/13	24/18	4/8	2.307 (chi-squared value)	0.315
SOFA score on ED arrival (median (Q1, Q3))	2 (1, 4)	2 (1, 4)	4 (2, 4)	20.052 (Kruskal-Wallis test)	※2
Anesthesia time (min) (median (Q1, Q3))	223 (181, 399)	212 (165, 278)	205 (174, 228)	1.808 (Kruskal-Wallis test)	0.405
Operation time (min) (median (Q1, Q3))	170 (125, 263)	150 (101, 216)	157 (101, 165)	4.499 (Kruskal-Wallis test)	0.106
Blood loss during operation (ml) (median (Q1, Q3))	238 (50, 504)	100 (20, 370)	261 (64, 402)	7.081 (Kruskal-Wallis test)	0.029
Blood transfusion during operation (ml) (median (Q1, Q3))	420 (0, 2430)	280 (0, 1560)	280 (0, 1338)	2.731 (Kruskal-Wallis test)	0.255
SOFA score on ICU admission (median (Q1, Q3))	7 (3, 10)	6 (3, 9)	8 (4, 11)	2.617 (Kruskal-Wallis test)	0.27
Duration of ICU stay (days) (median (Q1, Q3))	4.0 (3.0, 8.0)	4.0 (3.0, 8.0)	3.0 (2.8, 13.0)	0.027 (Kruskal-Wallis test)	0.987
Duration of hospital stay (days) (median (Q1, Q3))	18.0 (14.0, 37.0)	20.0 (13.0, 37.0)	31.0 (20.0, 42.8)	1.546 (Kruskal-Wallis test)	0.462
Medical costs ($) (median (Q1, Q3))	13,074 (8,549, 32,945)	15,201 (9,112, 29,134)	14,430 (9,053, 38,714)	0.106 (Kruskal-Wallis test)	0.949

Table 2 summarizes the causes of emergency operations and the surgical procedures performed. The proportion of endogenous causes was 73.3% in Group Y, 76.2% in Group M, and 91.7% in Group E, with Group E showing the highest rate, although the difference was not statistically significant. No laparoscopic surgeries were performed in Group Y, while no thoracotomies were conducted in Group E. Further details of the emergency surgeries are provided in Table 3.

**Table 2:** Causes of emergency operations and types of surgical procedures. *Others include necrotizing enterocolitis and superior mesenteric artery thrombosis.

Items	Group Y (n = 30)	Group M (n = 42)	Group E (n = 12)	Test statistic	p-value
Causes of emergency operations	30	42	12	1.714 (chi-squared value)	0.524
Endogenous	22 (73.3%)	32 (76.2%)	11 (91.7%)	14.135 (chi-squared value)	0.439
Perforation of digestive tract	8	12	5	Not analyzed	Not analyzed
Acute aortic dissection	6	4	0	Not analyzed	Not analyzed
Strangulated ileus	3	7	3	Not analyzed	Not analyzed
Internal hernia	2	1	0	Not analyzed	Not analyzed
Empyema	1	0	0	Not analyzed	Not analyzed
Aneurysmal rupture (thoracic, abdominal, others)	1	6	2	Not analyzed	Not analyzed
Idiopathic esophageal rupture	1	0	0	Not analyzed	Not analyzed
Others*	0	2	1	Not analyzed	Not analyzed
Exogenous	8 (26.7%)	10 (23.8%)	1 (8.3%)	0.950 (chi-squared value)	0.622
Blunt injury	6	9	1	Not analyzed	Not analyzed
Penetrating injury	2	1	0	Not analyzed	Not analyzed
Types of emergency operations	30	42	12	19.168 (chi-squared value)	0.159
Endogenous	22 (73.3%)	32 (76.2%)	11 (91.7%)	13.778 (chi-squared value)	0.032
Laparotomic	12	17	9	Not analyzed	Not analyzed
Laparoscopic	0	7	2	Not analyzed	Not analyzed
Thoracotomy	8	4	0	Not analyzed	Not analyzed
Others	2	4	0	Not analyzed	Not analyzed
Exogenous	8 (26.7%)	10 (23.8%)	1 (8.3%)	6.534 (chi-squared value)	0.366
Laparotomic	6	7	0	Not analyzed	Not analyzed
Laparoscopic	0	1	0	Not analyzed	Not analyzed
Thoracotomy	1	1	0	Not analyzed	Not analyzed
Others	1	1	1	Not analyzed	Not analyzed

**Table 3:** Details of emergency operations. * In Group Y, other procedures included partial ileal resection (n = 2), omental patch repair (n = 2), appendectomy (n = 1), Hartmann's operation (n = 1), and closure of a perforated lesion with omental patch repair (n = 1). In Group M, other procedures included partial ileal resection (n = 2), Hartmann’s operation (n = 2), omental patch repair (n = 1), closure of a perforated lesion with omental patch repair (n = 1), and omental patch repair with construction of an intestinal fistula (n = 1). In Group E, other procedures included ileocolic resection with colostomy (n = 1), colectomy with colostomy (n = 1), and closure of a perforated lesion with omental patch repair (n = 1). ** In Group M, other procedures included appendectomy (n = 1), colostomy (n = 1), and closure of a perforated lesion with omental patch repair (n = 1). *** In Group Y, other procedures included partial ileal resection with mesenteric repair (n = 1), partial ileal resection with repair of the descending colon and appendico-serosal layer (n = 1), and repair of the retroperitoneum (n = 1). In Group M, other procedures included closure of perforated lesions in the stomach, ascending colon, and transverse colon (n = 1), ileal resection (n = 1), and closure of a perforated ileum (n = 2). AAA: Abdominal aortic aneurysm; ADL: Activities of daily living; BI: Barthel Index; EVAR: Endovascular aneurysm repair; IIA: Internal iliac artery; SOFA: Sequential Organ Failure Assessment; TAE: Transarterial embolization; TEVAR: Thoracic endovascular aortic repair.

Items	Group Y (n = 30)	Group M (n = 42)	Group E (n = 12)
Endogenous	22	32	11
Laparotomic surgery	12	17	9
- Intraperitoneal drainage + other procedures*	7	7	3
- Surgical release of strangulated ileus and ileal resection	2	3	0
- Ileal resection	0	2	4
- Open surgical graft explantation for ruptured AAA	0	2	2
- Surgical release of strangulated ileus	1	1	0
- Colectomy	0	1	0
- Colectomy + colostomy	0	1	0
- Ileal resection + colostomy	1	0	0
- Gastrojejunostomy	1	0	0
Laparoscopic surgery	0	7	2
- Intraperitoneal drainage + other procedures**	0	3	0
- Surgical release of strangulated ileus + ileal resection	0	1	0
- Surgical release of strangulated ileus	0	1	0
- Ileal resection + femoral hernia repair	0	1	0
- Ileal resection	0	0	1
- Appendectomy	0	0	1
Thoracotomy	8	4	0
- Ascending aortic replacement	3	3	0
- Hemi-arch replacement	2	1	0
- Open-window thoracostomy for pleural empyema with fistula	1	0	0
- Ascending and aortic arch replacement + right common carotid arterial bypass	1	0	0
- Esophageal suturing + intrathoracic drainage + T-tube drainage + fundic patch	1	0	0
Others	2	4	0
- EVAR (including TEVAR)	1	3	0
- EVAR + IIA coil embolization	0	1	0
- Intrathoracic drainage + intraperitoneal drainage + omental patch repair	1	0	0
Exogenous	8	10	1
Laparotomic surgery	6	7	0
- Intraperitoneal drainage + other procedures***	3	4	0
- Distal pancreatectomy + hemostasis	1	0	0
- Ileal resection	1	1	0
- Closure of perforated ileum and hemostasis	1	0	0
- Closure of perforated omentum and puncture wound	0	1	0
- Colectomy	0	1	0
Laparoscopic surgery	0	1	0
- Ileal resection	0	1	0
Thoracotomy	1	1	0
- Right upper lobectomy + ligation of right subclavian artery	1	0	0
- Pleural suture of right upper lobe + ligation of intercostal artery	0	1	0
Others	1	1	1
- Laparotomic splenectomy + thoracotomic left lower lobectomy + 6th rib resection after TAE	1	0	0
- TEVAR	0	1	0
- EVAR + TAE	0	0	1

**Figure 1:**
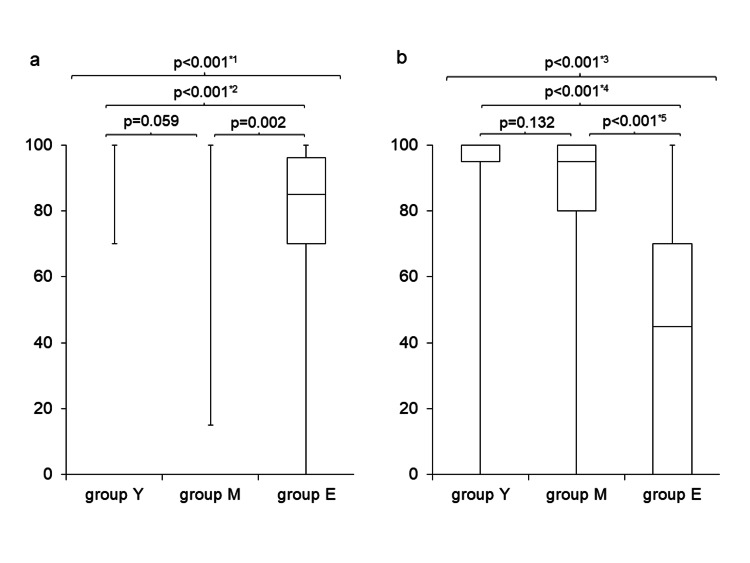
Comparisons of Barthel Index (BI). a. Preoperative BI Preoperative BI values in Groups Y, M, and E were 100 (100, 100), 100 (100, 100), and 85 (70, 96), respectively (data shown as median (Q1, Q3)). *1: p = 0.0001068; *2: p = 0.00001922 b. Postoperative BI at hospital discharge Postoperative BI values in Groups Y, M, and E were 100 (95, 100), 95 (80, 100), and 45 (0, 70), respectively (data shown as median (Q1, Q3)). *3: p = 0.0000001531; *4: p = 0.0000000304; *5: p = 0.0000027224

Figure 1 compares pre- and postoperative BI scores across the three groups, with postoperative BI assessed at hospital discharge.

Both pre- and postoperative BI scores in Group E were the lowest among the groups, showing significant differences. Additionally, postoperative BI significantly declined compared to preoperative scores in all three groups:

Group Y: preoperative BI: 100 (100, 100), postoperative BI: 100 (95, 100), p = 0.036

Group M: preoperative BI: 100 (100, 100), postoperative BI: 95 (80, 100), p = 0.038

Group E: preoperative BI: 85 (70, 96), postoperative BI: 45 (0, 70), p = 0.003

As shown in Table 1, no significant differences were observed in ICU or hospital stay duration among the three groups. Medical costs at our hospital were similar across the groups, with no significant differences (Table 1; $1 = 157.41 yen).

**Figure 2:**
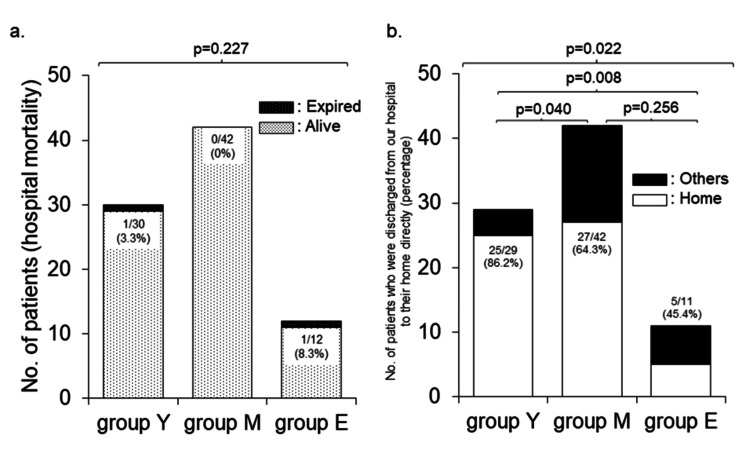
Comparisons of hospital mortality and patients discharged directly to home. a. Number of survivors and deceased patients (hospital mortality). b. Number and proportion of patients discharged directly to home.

Hospital mortality was highest in Group E, although the difference was not statistically significant (Figure 2a). In contrast, the proportion of patients discharged directly to their homes differed significantly among the groups, with Group E showing the lowest rate (Figure 2b).

## Discussion

This study found no significant differences in ICU and hospital stay durations, hospital mortality, or medical costs among the three groups, despite significant differences in SOFA scores upon ED arrival and intraoperative blood loss. These findings may reflect advances in perioperative management, including anesthesia and intensive care. However, preoperative ADL was significantly lower in Group E compared with the other groups. Postoperative ADL declined across all groups, with Group E showing the most pronounced deterioration. Additionally, the proportion of patients discharged directly home was significantly lower in Group E. These results suggest that emergency surgeries (abdominal, pulmonary, and vascular under general anesthesia) reduce ADL across all age groups, particularly in those aged 85 and older. Consequently, the total duration of required medical care, including rehabilitation, and overall medical costs are likely to increase in this population.

Emergency surgery often results in functional decline due to preoperative deconditioning and/or postoperative complications [6]. The loss of preoperative abilities such as mobility or independence in ADL is among the most feared postoperative outcomes for geriatric patients [12] and may necessitate caregiver support or discharge to a nursing facility [13]. This loss of independence affects quality of life and imposes significant economic and social burdens on families and society [14]. Importantly, age alone should not determine treatment suitability [15], ICU triage decisions [16], or be considered a reliable predictor of surgical risk [17].

In this study, no significant differences were observed in ICU or hospital stay durations or hospital mortality among the three groups, as previously described. However, ADL in Group E, already lower preoperatively, deteriorated more markedly than in the other groups. Our previous work has emphasized the importance of medical care, including intensive care, for elderly patients [18-20]. In those studies, we demonstrated that ADL following trauma was significantly lower in elderly patients (aged ≥80) requiring intensive care compared with younger trauma patients [20]. Similarly, Zattoni D et al. [21] reported that age ≥85 was significantly associated with functional decline following emergency abdominal surgery. Nagakawa K et al. [22] also found that pre-admission physical status was significantly worse in patients who became bedridden after emergency general surgery compared with those who maintained mobility, in a cohort aged over 75. The present findings are consistent with those reports. When patients over 85 years undergo emergency surgery, informed consent should include not only discussions of potential postoperative complications and mortality but also the substantial risk of functional decline and reduced postoperative ADL.

Although it remains unclear whether elderly patients can fully regain preoperative ADL after significant decline following emergency surgery, long-term rehabilitation is clearly required, and substantial medical costs are inevitable. In 2022, the annual medical cost per person aged ≥65 in Japan was estimated at $4,929 (1 USD = 157.41 yen), which is 3.7 times higher than that for individuals under 65 ($1,331), according to the Ministry of Health, Labour and Welfare [23]. Thus, managing healthcare costs for the elderly has become a pressing social issue. Increasing the number of older adults who remain healthy with preserved ADL may help address this challenge. Typically, maximal oxygen consumption decreases by approximately 10% per decade, and skeletal muscle mass declines at a similar rate between the ages of 60 and 70 [24]. However, research has shown that these declines can be mitigated through appropriate physical training [25]. Therefore, promoting frailty prevention, managing comorbidities, and maintaining high ADL levels in older adults are essential strategies for preserving postoperative function and reducing healthcare costs in this population.

This study has several limitations. It was a retrospective, single-institution analysis with a relatively small sample size. Only patients deemed capable of withstanding the physiological stress of emergency surgery were included. Postoperative complications were not evaluated; however, the absence of significant differences in ICU and hospital stay durations suggests that complication rates may not have differed substantially among the groups. Additionally, long-term postoperative outcomes were not assessed. Future research should examine long-term prognosis following emergency surgery, the role of rehabilitation in improving ADL, and the associated long-term medical costs in elderly patients.

## Conclusions

Emergency operations in super-elderly patients (≥85 years) may provide curative outcomes comparable to those in younger patients; however, the overall treatment period, including postoperative rehabilitation, is likely to be prolonged due to significantly reduced postoperative ADL.
